# SARS-CoV-2 pneumonia with subcutaneous emphysema, mediastinal emphysema, and pneumothorax: A case report

**DOI:** 10.1097/MD.0000000000020208

**Published:** 2020-05-15

**Authors:** Chunlin Xiang, Gang Wu

**Affiliations:** Department of Radiology, Tongji Hospital of Tongji Medical College of Huazhong University of Science and Technology, Wuhan, China.

**Keywords:** heart failure, pneumothorax, SARS-CoV-2, subcutaneous emphysema

## Abstract

**Introduction:**

Since the end of 2019, severe acute respiratory syndrome coronavirus 2 (SARS-CoV-2) infection has affected more than 1,000,000 population in the world. Subcutaneous emphysema and pneumothorax are uncommon complications of SARS-CoV-2 pneumonia. Herein, we describe a fatal case of SARS-CoV-2 pneumonia with subcutaneous emphysema and pneumothorax.

**Patient concerns:**

Subcutaneous emphysema was found in neck, bilateral chest walls, abdomen wall, groin area, and scrotum of a 67-year-old man. Extensive air-space opacities, subcutaneous emphysema and a small amount of pneumothorax were found in his chest X-ray scan. Echocardiography showed left ventricular enlargement with ejection fraction 20%.

**Diagnosis:**

This resident of Wuhan with laboratory-confirmed SARS-CoV-2 infection had chronic pulmonary and cardiac diseases. Liver dysfunction, myocardial injury, and coagulation disorder were suggested by laboratory findings. Pneumonia, subcutaneous emphysema, and pneumothorax were confirmed with chest X-ray. Heart failure was revealed by echocardiography.

**Interventions:**

He was transferred to intensive care unit, where invasive ventilation was used for him during the whole hospitalization. Prone position ventilation, vasoconstrictor, antibacteria, and antiviral therapy were given.

**Outcomes:**

He died on the twelfth day after admission.

**Conclusions:**

Subcutaneous emphysema and pneumothorax may occur in patients with SARS-CoV-2 pneumonia and chronic pulmonary disease. Chronic cardiac disease might be aggravated by SARS-CoV-2 infection, and develop heart failure.

## 1 Introduction

Since the end of 2019, severe acute respiratory syndrome coronavirus 2 (SARS-CoV-2) infection has affected more than 1,000,000 population, and resulted in thousands of deaths in the world. There are plenty of publications reporting the clinical features and outcomes of critically ill patients with SARS-CoV-2 infection.^[1,2]^ However, few publications reported in detail the rare complications of SARS-CoV-2 pneumonia including subcutaneous emphysema and pneumothorax. We here report a fatal case of SARS-CoV-2 infection with multiple rare complications. The patient has provided informed consent for publication of the case.

## 2 Case report

A 67-years-old male resident of Wuhan with laboratory-confirmed SARS-CoV-2 infection was admitted to the author's center on Feb 10. He had dyspnea for half a month, accompanied by fatigue and mild diarrhea. The patient had a history of coronary artery bypass, and chronic pulmonary diseases including obsolete pulmonary tuberculosis, chronic bronchitis, and emphysema. Body temperature and blood pressure were 37.2°C and 149/85 mm Hg, respectively at admission, while respiratory rate and pulse were normal. The breath sounds of both lungs were thick, and dry and wet rales could be heard at the lung base. The heart boundary was enlarged to the left and down, and murmurs could be heard.

The main abnormalities in laboratory findings at admission were as follows: glutamic-pyruvic transaminase (GPT) 63 U/L, albumin 32.8 g/L, lactate dehydrogenase (LDH) 535 U/L, leukocyte count 15.96 × 10^9^/L, neutrophil 12.86 × 10^9^/L, lymphocyte 2.04 × 10^9^/L, procalcitonin 0.10 ng/mL, D-dimer 3.53 μg/mL FEU, glucose 7.43 mmol/L, and C-reactive protein (CRP) 45.8 mg/L.

He was given high flow intranasal oxygen inhalation with oxygen concentration 100%. However, he still had obvious shortness of breath, 87% pulse oxygen saturation (SpO2), and poor consciousness. Thus he was transferred to intensive care unit (ICU), where invasive ventilation was used for him during the whole hospitalization, maintaining SpO2 at about 95%. Other treatment included prone position ventilation, vasoconstrictor, antibacteria, and antiviral therapy. His blood pressure could be maintained at about 110/60 mm Hg with noradrenaline (NE) pumped in.

Subcutaneous emphysema was found in his left neck 5 days after admission (Feb 15), and the area of subcutaneous emphysema gradually increased. Five days later, extensive subcutaneous emphysema could be seen in the neck, bilateral chest walls, abdomen wall, bilateral groin area, and scrotum.

**Figure 1:**
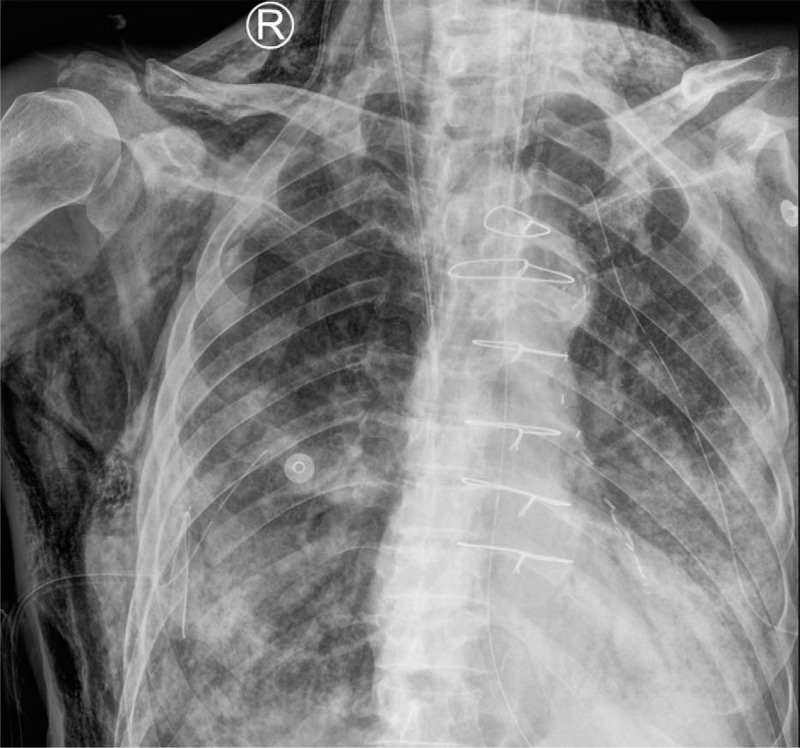
A 67-yr-old male resident of Wuhan with chronic cardiac and pulmonary diseases had dyspnea, accompanied by fatigue, and mild diarrhea. RT-PCR result was positive for SARS-CoV-2. He had a history of coronary artery bypass. He underwent invasive ventilation for correcting hypoxemia. Mobile X-ray was used for assessing SARS-CoV-2 infection instead of CT. The chest radiograph revealed extensive air-space opacities in bilateral lungs, with lower lung involvement more serious than upper lung. Extensive subcutaneous emphysema in neck and chest wall could be easily seen. Mediastinal emphysema and a small amount of pneumothorax were also identified, as well as enlargement of the left ventricle. The patients died of respiratory and heart failure 2 d after this scan.

He underwent mobile X-ray 10 days after admission (Feb 20) for assessing SARS-CoV-2 pneumonia, as chest CT was unavailable for him. The chest radiograph revealed extensive air-space opacities in bilateral lungs, with lower lung involvement more serious than upper lung (Fig. 1). Subcutaneous emphysema, mediastinal emphysema, and a small amount of pneumothorax on both sides (10–20% compression of lung) could also be seen. Chest closed drainage was thus performed for him immediately.

The main abnormalities in laboratory findings during his hospitalization were as follows: leukocyte count 3.12 to 20.44 × 10^9^/L, neutrophil 2.94 to 19.24 × 10^9^/L, lymphocyte 0.18 to 0.32 × 10^9^/L, hemoglobin 109.0 to 131.0 g/L, platelet count 40.0 to 140.0 × 10^9^/L, potassium 5.09 to 6.20 mmol/L, calcium 1.78 to 1.94 mmol/L, total protein 46.7 to 59.4 g/L, albumin 21.1 to 30.3 g/L, GPT 55 to 76 U/L, glutamic oxaloacetic transaminase (GOT) 102 to 104 U/L, total bilirubin 20.4 to 22.4 μmol/L, direct bilirubin 11.2 to 13.8 μmol/L, urea 12.10 to 16.00 mmol/L, hypersensitive CRP 202.0 to 216.8 mg/L, D-dimer 1.61 to >21.00 μg/mL FEU, prothrombin time 15.9 to 18.1 seconds, prothrombin activity 55.0% to 71.0%, international normalized ratio 1.24 to 1.49, fibrinogen 3.95 to 5.55 g/L, activated partial thromboplastin time (APTT) 42.9 to 80.9 seconds, fibrinogen degradation products 6.7 to 9.3 μg/mL, creatine kinase MB isoenzyme 4.3 to 208.3 ng/mL, myoglobin 198.2 to >1200.0 ng/mL, high sensitive cardiac troponin I 39.5 to 361.6 pg/mL, interleukin-6 472.20 pg/mL. The platelet, lymphocyte, and albumin continued to decrease until death, while GPT, GOT, APTT, and myoglobin continued to increase.

Sinus bradycardia (heart rate 30–45 bpm) was observed 11 days after admission (Feb 21). Echocardiography was performed immediately, showing left ventricular enlargement with ejection fraction 20%. Coagulation function was even worse for him, and a small amount of blood oozed from nose and mouth during prone position. There was still gas emerging from the thoracic drainage tube. Extensive subcutaneous emphysema was even worse than before. As his blood pressure dropped to 78/45 mm Hg, NE was increased to 1.5 μg/kg min, accompanied with rapid fluid infusion. However, the blood pressure and heart rate did not rise. The increase of lactate (up to 4.0 mmol/L) suggested aggravation of respiratory failure.

The heart rate slowed down to 20 bpm, and blood pressure could not be detected on the twelfth day after admission (Feb 22). Adrenaline, noradrenaline, and dobutamine were immediately given. However, the heart rate and respiration stopped, and the ECG showed a straight line, and clinical death was declared.

## 3 Discussion

This patient with chronic obstructive pulmonary disease (COPD) and SARS-CoV-2 pneumonia underwent invasive ventilation for correcting hypoxemia. Unfortunately, subcutaneous emphysema and pneumothorax occurred and aggravated respiratory failure. The alveoli may be prone to rupture due to diffuse alveolar injury caused by SARS-CoV-2.^[3]^ Alveolar rupture caused air leakage and interstitial emphysema. A recent case report of a patient without invasive ventilation indicated that the mediastinal emphysema and pneumothorax may be related to SARS-CoV-2 infection.^[4]^ Because of the long-term increase of alveolar pressure, the decrease of alveolar capillary blood supply, and the impairment of lung tissue nutrition in COPD,^[5]^ the elasticity and tolerance of alveolar wall are weakened. Thus SARS-CoV-2 pneumonia in patients with COPD is more likely to cause spontaneous pneumothorax.

Pneumothorax, mediastinal emphysema, and subcutaneous emphysema are well-known complications of mechanical ventilation that is an important life support treatment for critical patients.^[6]^ During mechanical ventilation, the pressure in the respiratory tract is very high, which can increase the pressure gradient between alveoli and surrounding tissues, leading to the rupture of alveoli and the formation of interstitial emphysema. At the same time, because the average pressure in the mediastinum is lower than that in the surrounding pulmonary parenchyma, the gas enters the mediastinum, causing pneumomediastinum.^[7]^ In COPD patients, due to the increase of small airway resistance, positive pressure of the ventilator will send gas to the pulmonary bullae when inhaled. When exhaled, the gas in the pulmonary bullae is not easy to exhale, and the internal transmural pressure of the pulmonary bullae increases, resulting in rupture and pneumothorax. Improper operation in the process of endotracheal intubation may cause damage to the tracheal wall and further lead to subcutaneous emphysema. The airway of elderly patients with COPD is more vulnerable to tracheal intubation than that of normal people. It is worth noting that this patient had a history of thoracotomy. We speculate that previous thoracotomy is also a factor leading to the extensive subcutaneous emphysema in this patient.

Pneumothorax, mediastinal emphysema, and subcutaneous emphysema related to the use of ventilator can further aggravate the respiratory dysfunction. Therefore, patients using ventilator need to have a dynamic review of chest radiograph in order to find these complications in the early stage.^[8]^ SARS-CoV-2 patients with COPD and invasive ventilation are more likely to develop pneumothorax and subcutaneous emphysema, which will aggravate respiratory failure. Physicians should keep in mind of this point, and give repeated mobile X-ray scans to such patients.

This patient had chronic cardiac illness, which seemed to be aggravated by SARS-CoV-2 infection. Elevated level of markers of myocardial injury was observed in this patient. Heart failure was validated by echocardiography. The patient's blood pressure continued to drop until death. Heart failure and shock may be the main causes of death.

In conclusion, pneumothorax, mediastinal emphysema, and subcutaneous emphysema may occur in patients with SARS-CoV-2 infection. Chronic cardiac and pulmonary disease correlated with poor outcome of SARS-CoV-2 pneumonia.

## Author contributions

**Conceptualization:** Chunlin Xiang, Gang Wu.

**Data curation:** Chunlin Xiang, Gang Wu.

**Formal analysis:** Chunlin Xiang, Gang Wu.

**Funding acquisition:** Gang Wu.

**Investigation:** Chunlin Xiang, Gang Wu.

**Methodology:** Chunlin Xiang, Gang Wu.

**Project administration:** Gang Wu.

**Resources:** Chunlin Xiang, Gang Wu.

**Software:** Chunlin Xiang, Gang Wu.

**Supervision:** Chunlin Xiang, Gang Wu.

**Validation:** Chunlin Xiang, Gang Wu.

**Visualization:** Gang Wu.

**Writing – original draft:** Chunlin Xiang, Gang Wu.

**Writing – review & editing:** Gang Wu.
